# IUC23223-77 Triplet therapy in metastatic hormone-sensitive prostate cancer in Asian patients

**DOI:** 10.1093/oncolo/oyaf248.035

**Published:** 2025-08-29

**Authors:** A Zakirov, F Djuraev, O Raxmonov, A Jumaev

**Affiliations:** Tashkent Medical Park by Urologic Complex, Uzbekistan; Tashkent Medical Park by Urologic Complex, Uzbekistan; Tashkent Medical Park by Urologic Complex, Uzbekistan; Tashkent Medical Park by Urologic Complex, Uzbekistan

## Abstract

**Background:**

Two randomized trials demonstrated a survival benefit of triplet therapy, androgen deprivation therapy plus androgen receptor pathway inhibitor plus docetaxel over doublet therapy (ADT plus docetaxel), thus changing treatment strategies in metastatic hormone-sensitive prostate cancer (mHSPC).

**Methods:**

We conducted the real-world analysis comprising 92 mHSPC patients from 3 Tashkent medical centers, among them 75% of patients received abiraterone and 16.5% daroluramide treatment. Baseline characteristics and clinical parameters during triplet therapy were documented.

**Results:**

Of them, 78.2% patients with synchronous and 21.8% with metachronous disease were included. 82.6% had high-volume disease diagnosed by conventional imaging (47.8%) or PSMA PET-CT (52.2%). While docetaxel and ARPI were administered consistent with pivotal trials, prednisolone, prophylactic gCSF and osteoprotective agents were not applied according to guidelines in 31.5%, 43,5%, and 25% of patients, respectively. Importantly, a nonsimultaneous onset of chemotherapy and ARPI, performed in 51% of patients, was associated with significantly worse treatment response (*P* = .015, HR 0.245). Starting ARPI before chemotherapy was associated with a significantly higher probability for progression (*P* = .023, HR 15.781) than vice versa. Strikingly, 15.2% (abiraterone) and 25.0% (darolutamide) low-volume patients, as well as 14.1% (abiraterone) and 20.6% (darolutamide) metachronous patients, received triplet therapy. Adverse events (AE) occurred in 60.8% with grades 3 to 5 in 15% of patients without age-related differences. All patients achieved a PSA decline of 99% and imaging response was confirmed in 88% of abiraterone and 75% of daroluramide patients.

**Conclusions:**

Triplet therapy arrived in clinical practice primarily for synchronous high-volume mHSPC. Regardless of the selected therapy regimen, treatment is highly effective and tolerable. Preferably, therapy should be administered simultaneously; however, if not possible, chemotherapy should be started first

